# Spontaneous and training-induced cortical plasticity in MD patients: Hints from lateral masking

**DOI:** 10.1038/s41598-017-18261-6

**Published:** 2018-01-08

**Authors:** Marcello Maniglia, Vincent Soler, Benoit Cottereau, Yves Trotter

**Affiliations:** 1 UC Riverside, Riverside, California, USA; 2 Université de Toulouse-UPS, Centre de Recherche Cerveau et Cognition, Toulouse, France; 3 Centre National de la Recherche Scientifique, Toulouse Cedex, France; 4 Unité de rétine, consultation d’ophtalmologie, hôpital Pierre-Paul-Riquet, CHU de Toulouse, place Baylac, 31059 Toulouse cedex, France

## Abstract

Macular degeneration (MD) affects central vision and represents the leading cause of visual diseases in elderly population worldwide. As a consequence of central vision loss, MD patients develop a preferred retinal locus (PRL), an eccentric fixation point that replaces the fovea. Here, our aim was to determine whether and to what extent spontaneous plasticity takes place in the cortical regions formerly responding to central vision and whether a visual training based on perceptual learning (PL) can boost this plasticity within the PRL area. Spontaneous and PL-induced cortical plasticity were characterized by using lateral masking, a contrast sensitivity modulation induced by collinear flankers. This configuration is known to be sensitive to neural plasticity and underlies several rehabilitation trainings. Results in a group of 4 MD patients showed that collinear facilitation was similar to what observed in age- and eccentricity-matched controls. However, MD patients exhibited significantly reduced collinear inhibition, a sign of neural plasticity, consistent with the hypothesis of partial cortical reorganization. Three AMD patients from the same group showed a further reduction of inhibition after training, but not controls. This result suggests that PL might further boost neural plasticity, opening promising perspectives for the development of rehabilitation protocols for MD patients.

## Introduction

**Figure 1:**
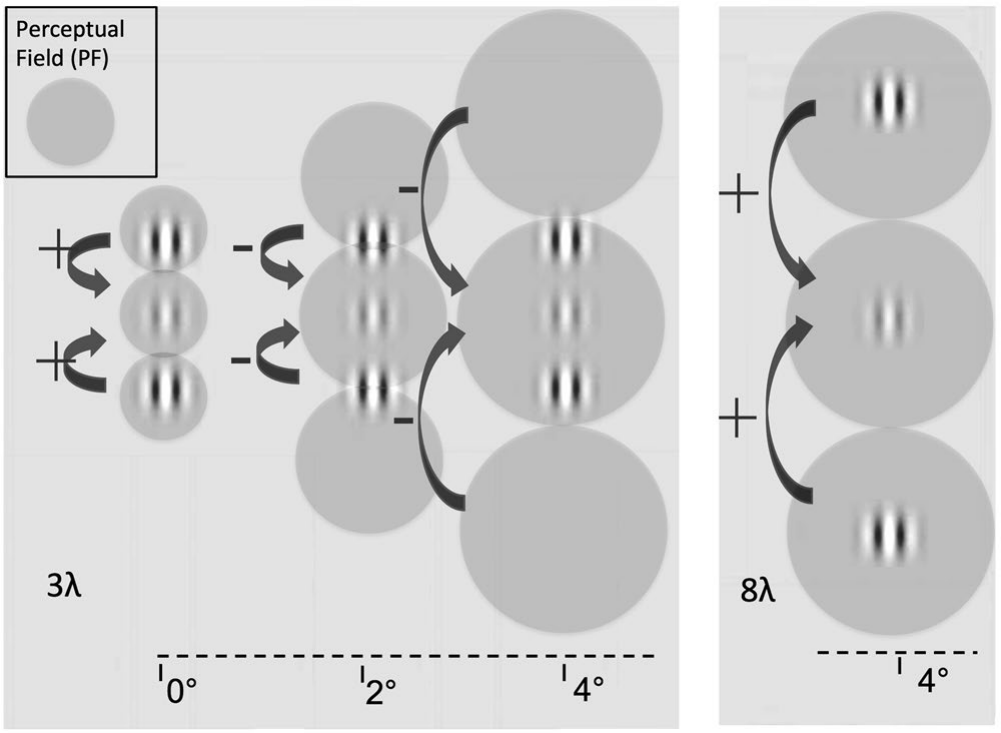
Schematic of the interaction between perceptual field (PF) size and collinear configuration as a function of eccentricity. X-axis represents eccentricity from central fixation. In foveal vision, the standard target-to-flankers distance that induces facilitation is 3λ (i.e., 3 times the wavelength of the Gabor patches of the configuration). In the present study, the spatial frequency was kept fixed at 1 cpd, therefore λ = 1 cpd. In this case, this distance also corresponds to the PF size and facilitation is considered as a between-PFs effect. When the same stimulus is presented in peripheral vision, the PF size increases, so that the 3λ configuration elicits within-PF, collinear inhibition. In order to restore facilitation, flankers must be placed outside the PF. Previous studies^19–21^ showed that this critical distance is 8λ at an eccentricity of 4°.

Macular degeneration (MD) is a visual pathology that affects central vision and represents the leading cause of blindness in the elderly population^1^. Loss of central vision strongly impairs everyday life activities, such as locomotion, reading or face recognition. MD patients usually develop an eccentric retinal spot outside the scotoma, the preferred retinal locus (PRL), as a new fixation point^2^. The consequences of the PRL development on visual perception remain unclear. A recent study^3^ measured crowding, the difficulty to identify peripheral targets when surrounded by flanking elements^4^ in MD patients. This study showed that the shape of the crowding zone for targets presented at the PRL resembled that of the fovea rather than the periphery. This result could reflect spontaneous reorganization in cortical regions connected to the PRL, most likely in early visual areas^4^. However, other studies found that performances in the PRL did not differ from those usually observed in normal peripheral vision^5,6^. This discrepancy challenges the extent of spontaneous reorganization in MD patients. Perceptual learning (PL) has recently gained considerable attention for its ability to improve sensory performances in normal and clinical populations^7^. In the last years, several studies have shown that this technique can be used to improve, to some extent, residual peripheral vision in MD patients^8–13^. However, in most of these studies, improvements were small and remained mostly specific to the trained task, with little transfer to other visual functions, in contrast to what was observed in other clinical populations (e.g., Amblyopia^14^, Presbyopia^15^ or Myopia^16^). More recently, MD patients were trained using a lateral masking paradigm^9^. In this configuration, a low contrast central target (a Gabor patch) is flanked collinearly by two elements with the same spatial frequency and orientation. Depending on the target-to-flankers separation, lateral masking can lead either to collinear inhibition, a decrease of contrast sensitivity, or to facilitation, an increase of contrast sensitivity for the central target^17^. The separation within which the flankers induce suppression provides an estimation of the perceptual field (PF), the psychophysical equivalent of the classical physiological receptive field (CRF)^18^. In this context, the suppression effect is considered as a within-PF effect, while collinear facilitation is a between-PFs effect^19^. Recent studies have shown that this effect exists outside foveal vision, with the facilitatory target-to-flankers separation (and consequently, the PF size) increasing with eccentricity, most likely following the cortical magnification factor (see Fig. 1).

This distinction between foveal and peripheral collinear facilitation is of critical importance in MD patients. Indeed, evidence for facilitation in the PRL at shorter separations than in healthy peripheral vision would be consistent with the idea of a spontaneous cortical reorganization after retinal lesion, similar to what is reported in crowding^4^. While they share similar features, such as the increase in magnitude with eccentricity, and resemblance between target and flanking elements^17,22^, there is no consensus in the literature regarding the relationship between crowding and lateral masking^23^. Consequently, the evidence for spontaneous cortical reorganization in reshaping the crowding zone does not necessarily mean that the neural substrates underlying cortical inhibition are equally affected.

Horizontal connections between units sharing similar orientation and spatial frequency tunings in early visual cortex^24–26^ are known to be sensitive to PL-induced plasticity as suggested by lateral masking paradigm^27^. Enhancing these first stages improves in turn higher level visual processing that can rely on more efficient inputs after training^15,21^.

The question is still open whether collinear facilitation exists in MD patients’ PRL and whether it can be modulated through PL, as reported for other clinical populations^14^. Indeed, a previous study did not find collinear facilitation in MD patients^28^, possibly because given the tested target-to-flankers distance (4λ), the flankers remained within the inhibitory region of the PF. In the present paper, we aimed at addressing these questions in a small sample of AMD patients (and age-matched controls) using a lateral masking configuration as a baseline measure for spontaneous cortical reorganization (Experiment 1) and as a training configuration for guided cortical reorganization (Experiment 2). Previous PL studies based on collinear facilitation showed a reduction of inhibition after training^21,27^, an effect attributed to neural plasticity in early visual areas^24–26^. Consequently, reduced inhibition at baseline would be consistent with the hypothesis of spontaneous cortical reorganization similar, to what was observed in previous studies after training.

## Method

In Experiment 1, we measured collinear facilitation (and inhibition) over a range of target-to-flankers distances in the PRL of 5 MD patients and 5 age- and eccentricity-matched controls. In Experiment 2 we trained a subgroup of participants from Experiment 1 (4 MD patients and 3 controls) to test whether facilitation and/or inhibition can be modulated through practice as observed in previous studies^21,27^. Because training based on PL are very long, a small sample size is not uncommon in PL studies. Nonetheless, a number of studies showed that reliable and robust results could still be obtained by comparing perfomances before and after training^29–34^.

Stimuli were three aligned Gabor patches which were displayed either in a collinear (flankers having the same local orientation as the target) or in an orthogonal (flankers having an orthogonal orientation with respect to the target i.e., a 90 deg offset) configuration (see^20^). The stimuli were aligned vertically for all participants except for MD3 and her matched control, for whom they were oriented horizontally, because of the shape of MD3’s scotoma. Each Gabor patch had a spatial frequency of 1 cpd. In a previous study, we showed that this frequency maximizes peripheral facilitation magnitude^35^. The target-to-flanker separations were 3λ, 4λ, 6λ and 8λ. Stimuli were displayed on a 17″ Dell M770 CRT monitor with a resolution of 1024 × 768 pixels, a refresh rate of 60 Hz and mean luminance of 47.6 cd/m^2^. Stimuli were generated with Matlab Psychtoolbox^36,37^. Each pixel subtended 2.14 arcmin. A digital-to-analogue converter (Bits#, Cambridge Research Systems, Cambridge UK) was used to increase the dynamic contrast range (13-bit luminance resolution). A 12-bit gamma-corrected lookup table (LUT) was used to linearize the monitor. Experiments were carried on at the Centre de la Retine, Hôpital Pierre-Paul Riquet, Purpan Hospital, Toulouse (France).

### Participants

Patients and controls’ information is detailed in Table 1. Importantly, in order to keep our sample of patients homogeneous, we only included in the study patients with a single and well-defined PRL. MD patients were instructed to fixate with their PRL the center of the screen where a fixation point was always present. They were asked to adapt their head position in order to fixate as naturally as possible. All participants were volunteers and gave written informed consent prior to their inclusion in the study. They received an allowance for their participation. This study was conducted in accordance with the Declaration of Helsinki (1964). The experimental protocol was approved by a national ethical committee before the beginning of the study (CPP, Comité de Protection des Personnes, protocole 13018–14/04/2014). Four patients (MD1, MD2, MD3 and MD4) and 3 controls (C1, C2 and C4) took part in Experiment 2. MD5, C3 and C5 were not able to participate in the training study for logistical reasons.

**Table 1:** Summary of characteristics of participants in the Experiments. PRL coordinates with SD for the three consecutive measures and scotoma size correspond to the tested eye. Clinical annotation: OS = left eye, OD = right eye.

**Participant**	**Sex**	**Age at the onset (years)**	**Time since the onset (years)**	**Year of birth**	**Diagnosis**	**Scotoma size**	**PRL coordinates (x axis)**	**PRL coordinates (y axis)**	**Tested eye**
MD1	M	61	7	1947	AMD	18.4°15.8°	8.15° +/− 0.1°	0.1° +/− 0.23°	OS
MD2	F	59	7	1949	AMD	17° × 23.4°	5.43° +/− 1.3°	1.14° +/− 0.7°	OS
MD3	F	82	4	1929	AMD	25.3° × 20.2°	2.6° +/− 0.14°	9.12° +/− 0.7°	OD
MD4	F	53	7	1958	Atrophic MD	12.1° × 7.4°	7.95° +/− 0.1°	1.6° +/− 0.3°	OS
MD5	F	77	3	1935	AMD	11.7° × 13.3°	9.25° +/− 1.6°	4.8° +/− 0.65°	OD
C1	F	—	—	1949		—	—	—	OS
C2	F	—	—	1951		—	—	—	OS
C3	F	—	—	1930		—	—	—	OD
C4	M	—	—	1955		—	—	—	OS
C5	M	—	—	1945		—	—	—	OD

#### Inclusion criteria

Absolute central binocular scotoma (as measured by 30° and 12° central visual fields analysed with Octopus® 300 perimeter; Haag-Streit, Köniz, Switzerland), age >60, residual vision in both eye < 2/10, presence of a single PRL (as detected with OCT measurement, see the ‘*PRL location’* section). Exclusion criteria: Concomitant presence of other visual diseases (cataract, retinal detachment, glaucoma), cognitive impairment (Mini-Mental State Examination (MMSE) score <25), monocular MD.

### PRL localization

**Figure 2:**
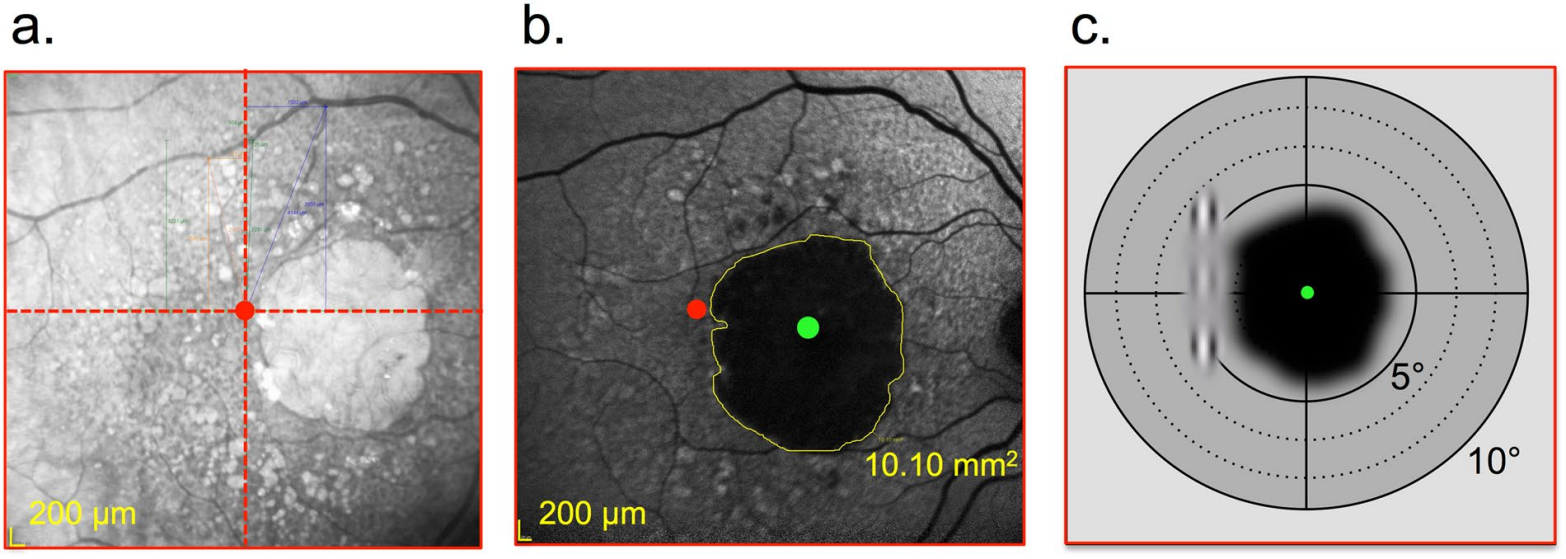
OCT procedure for PRL localization. (**a**) Retinal analysis via OCT: Infrared 2D macular image of the ‘cross fixation’ acquisition showing the macular atrophy (ligther rounded area). In this image, the center of the red dashed cross corresponds to the preferred retinal locus (PRL), the eccentric fixation point replacing the fovea. (**b**) Autofluorescence imaging with the location of the anatomical fovea (green dot) and the PRL position (red dot). (**c**) Schematic retinal projection of the stimulus on the PRL location close to the scotoma (dark area); green dot is the location of the fovea.

**Figure 3:**
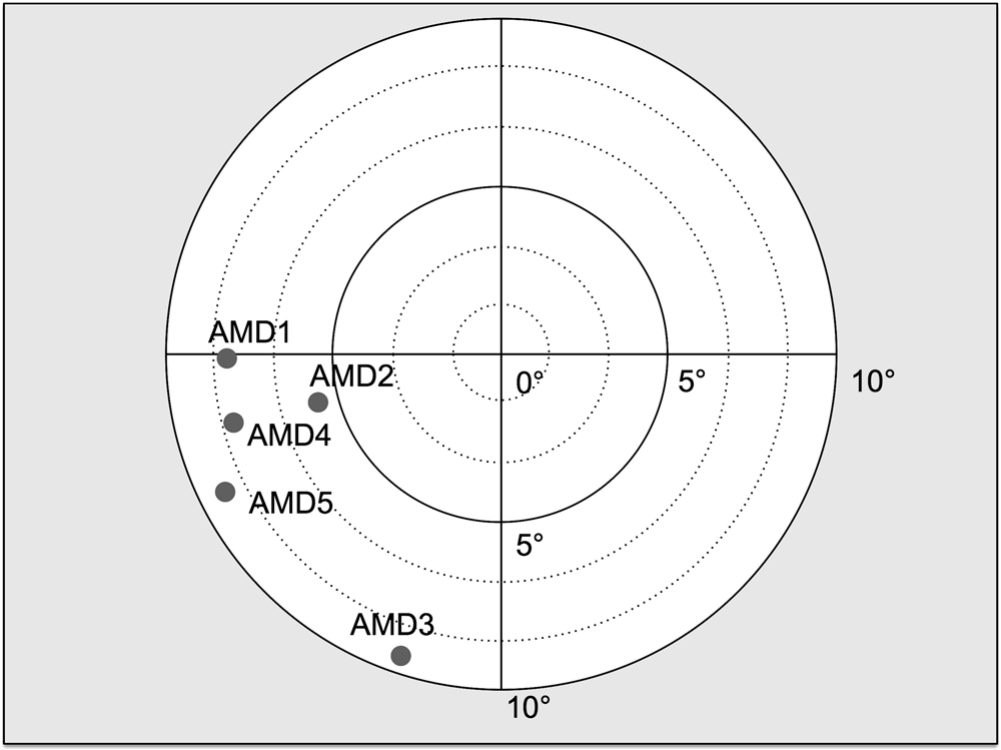
PRL position for participants in Experiment 1. Coordinates were averaged across three consecutive OCT measurements (see the ‘OCT method’ section).

For each MD patient, we determined the PRL position using Spectral-Domain Optical Coherence Tomography (Spectralis OCT, Heildelberg Enginering, Heidelberg, Germany). First, high resolution scans of the retinal fundus, centered on the atrophic macular area, were acquired via OCT in order to localize the fovea (Fig. 2a). Three additional landmarks (i.e., crossing blood vessels) were identified on the infrared image of the macula and their coordinates were measured with respect to the fovea. In a successive acquisition, patients were asked to fixate with their PRL the central fixation point presented by the OCT system while high speed X-line OCT B-scans were acquired (Fig. 2b). To avoid the inclusion of patients with more than one PRL, this operation was repeated at least three times, and between each acquisition, the OCT system was defocalized while the patient was asked to fixate elsewhere. The same acquisition protocol was systematically conducted at each examination. As noted previously, patients with more than one PRL were excluded from the study. Afterwards, the distance between the PRL and the fovea was derived by comparing its position with respect to the three landmarks (Table 1 and Fig. 3). The small SD range of the three separate measures of PRL coordinates (Table 1) is a further indication that the patients were using a single PRL for fixation tasks.

### Procedure

**Figure 4:**
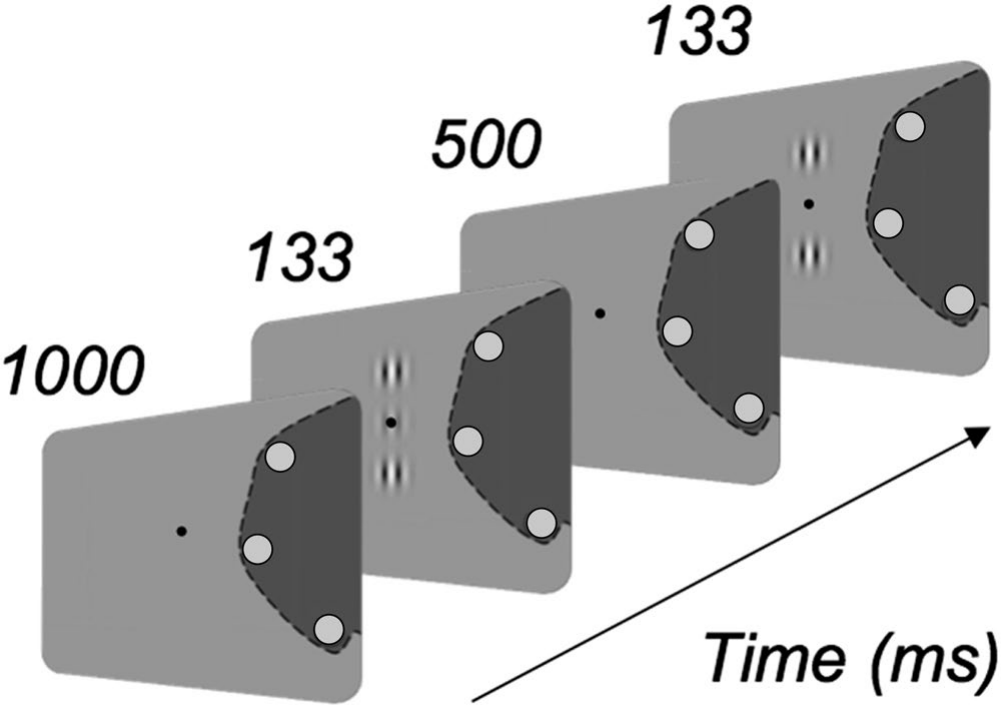
Experimental paradigm. Patients were instructed to fixate monocularly the point in the center of the screen with their PRL. In order to maximize fixation stablity, we displayed three additional red disks along the internal border of their scotoma (dark grey on the figure), selected individually for each patient within 2 deg from the border of the absolute scotoma. Patients had to adjust their eye position so that these points remained invisible. After 1000 ms, three vertically presented Gabors, either in a collinear (or orthogonal) configuration, appeared at this central position for 133 ms with an Inter Stimulus Interval (ISI) of 500 ms. In the collinear configuration, the flankers were presented above and below the target with the same (vertical) local orientation. In the orthogonal configuration, the flankers appeared above and below the target but their local orientation was horizontal (90° offset with respect to the target). Patients had to report which interval contained the target. Each control participant had to fixate foveally while the configuration was displayed at the eccentricity used in his/her paired MD patient.

In Experiment 1, we tested eight conditions per participant, one for each target-to-flankers distance (3λ, 4λ, 6λ, 8λ) and orientation (collinear and orthogonal). MD patients performed the task monocularly with their dominant eye. They fixated with their PRL while the configuration was presented at the centre of the screen. A fixation point was always present to indicate the center of the configuration. Patients were instructed to keep their fixation on the central dot with their PRL and to use the three discs to adjust their gaze on the central dot (Fig. 4). A similar approach was already taken in previous studies that used visual aids to stabilize fixation in this clinical population^38^. Each control participant was assigned to a patient of similar age and trained at the eccentricity corresponding to the PRL position of his/her paired MD patient. Control participants had to fixate a central dot while the position of the testing configuration was randomized along the *x* axis. Position along the y-axis was fixed. Participants had to perform a contrast detection task with a temporal-2AFC (two alternative forced choice) procedure, with one interval containing both target and flankers and the other interval containing only the flankers (see Fig. 4). Each interval lasted 133 ms and the ISI was 500 ms. Participants had to report in which interval they detected the target. Target contrast varied according to a 3down/1up staircase, in which three consecutive correct responses reduced the target contrast of 0.1 log units and each wrong response increased the contrast of the same amount. The staircase terminated after 120 trials or 14 reversals. Contrast thresholds (Michelson contrast), corresponding to 79% of correct detection, were estimated from the algebric mean of the last 6 reversals. Acoustic signals (50 ms tone of 1000 Hz) were provided to signal the beginning of each interval and as a feedback for uncorrect responses (50 ms tone of 500 Hz). Participants sat in a dark room at a distance of 57 cm from the screen. Testings were performed monocularly. The testing configuration was always vertical (see Fig. 4), except for MD3 and her corresponding control, for whom the global and local orientation of the triplet was horizontal. This was due to the position of the PRL and the shape of the scotoma of MD3 that would have made it difficult to allow for the three Gabor patches to be visible all the time in a horizontal configuration. All 8 conditions (4 target-to-flankers separations x2 orientations) were performed within a single day. In Experiment 2, participants were trained for 12 sessions (4 blocks per day, 3 sessions per week) on the same configuration as in Experiment 1. Target to flankers distances were varied between blocks in each daily session, starting from the largest separation (8λ, 6λ, 4λ, 3λ). Spatial frequency of the stimuli was 1cpd^34^. Participants were trained only with the collinear configuration and tested on the orthogonal configuration before and after the training. Before each experiment, we ran a practice block of 15 trials to ensure that the patients were able to see the two flankers while fixating in the center of the screen with their PRL. All participants (patients and controls) reported that both the target and the flankers were clearly visible under these experimental conditions.

### Statistical analyses

Given the small sample of participants in the Experiments, we conducted non-parametric statistical analyses. In particular, to explore the main effects, we used an Aligned Rank Transformation (ART) as described by Wobbrock, Findlater, Gergle and Higgins^39^ with the r package ARTool^40^. To test for interactions, we used a non-parametric ART test for interactions in designs with repeated measures as described by Beasley & Zumbo^41^ with the r package “npIntFactRep”^42^. However, while this last package permits to test effects for mixed models with one ‘*within’* and one ‘*between’* factors, it does not allow for testing interactions with more than one ‘*within’* factors, hence we only ran interaction tests for the Threshold elevation data. Post-hoc tests on the main effects were performed using linear models of the aligned data, as reported in Wobbrock *et al*.^39^. Finally, post-hoc tests on interactions were performed using differences of differences as suggested by Kay (supplementary documents in^40^). Indeed, directly comparing levels of factors in a non-parametric model is not a proper way of dealing with post hocs tests on interaction, and it is rather preferred to compare differences of differences, e.g., A-B|C *vs* A-B|D, where A and B are two levels of one factor and C and D are two levels of the other factor. Of note, ART tests do not allow for post-hoc tests on interactions when more than one within factor is present. These post-hoc tests were conducted using packages ARTool and phia in r.

## Results

### Pre training results (Experiment 1)

#### Contrast thresholds

**Figure 5:**
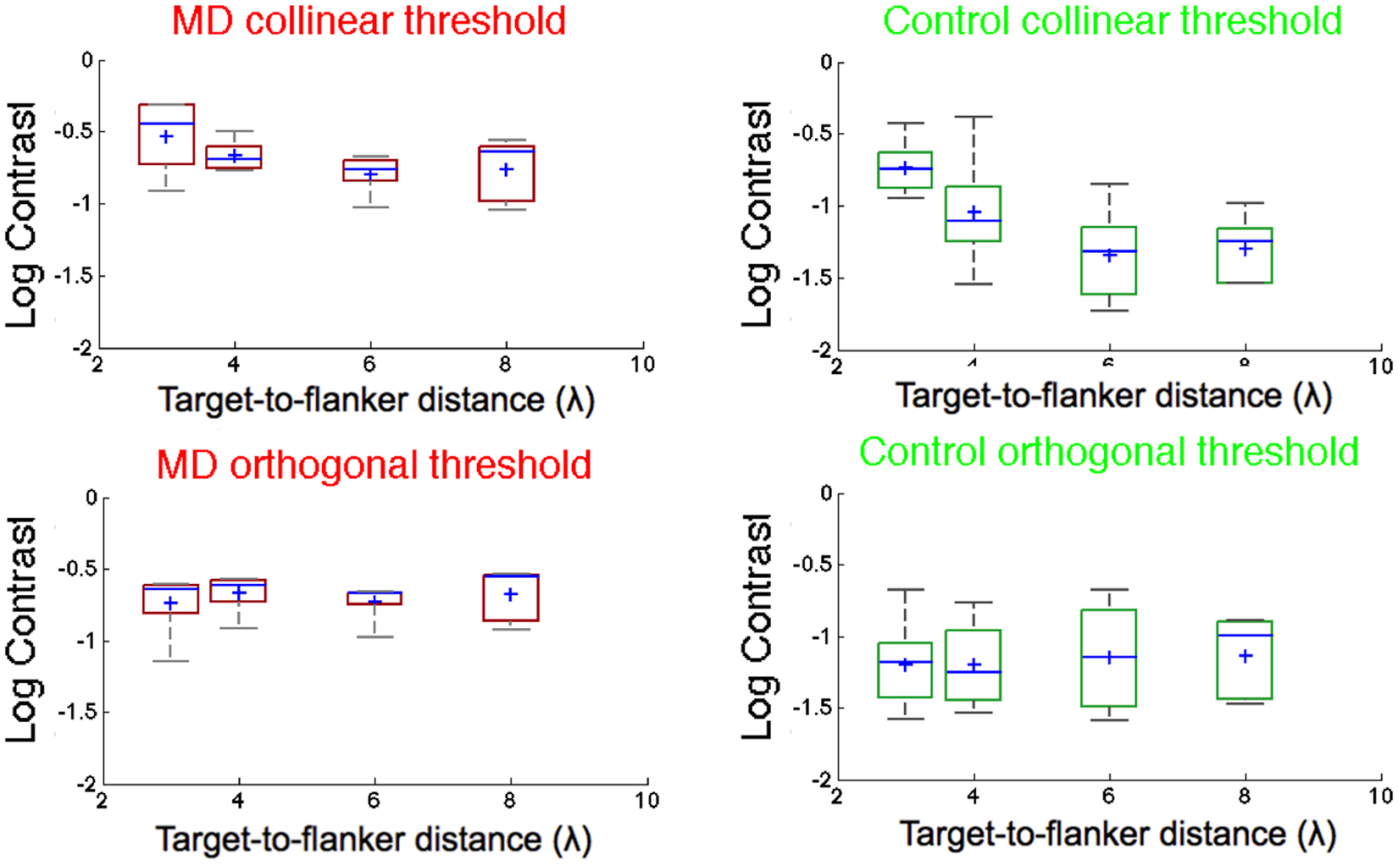
Box-and-whisker diagrams for collinear and orthogonal contrast thresholds (Michelson contrast) for MD (red boxes) and Control participants (green boxes) as a function of the target-to-flankers distances (λ). In each boxplot, the central mark is the median. The edges of the box are the 25th and 75th percentiles and the whiskers are the interquartile range (i.e., Q3–Q1). The black circles represent the mean and the vertical bars are +/− SEM.

Results for Experiment 1 are presented in Fig. 5 and Table 2. A Mixed Model ART on the contrast thresholds (Michelson contrast) of between-subjects factor Group (MD participants *vs* Controls) and within subjects factors Orientation (Collinear *vs* Orthogonal) and Target-to-flankers distances (3λ, 4λ, 6λ, 8λ) showed a significant main effect of Group (*F*_1,8_ = 10.604, *p* = 0.011) and of Target-to-flankers distances (*F*_3,56_ = 9.77, *p* < 0.001). As reported earlier, we could not test for the interaction effects (see Statistical analyses section). Post-hoc analysis, in the form of differences of differences, showed that the difference between collinear and orthogonal thresholds was significantly different for 3λ with respect to 8λ (p < 0.001, iso-ortho|3λ *vs* iso-ortho| 8λ) and for 3λ *vs* 6λ (p < 0.001, iso-ortho|3λ *vs* iso-ortho| 6λ).

**Table 2:** Individual contrast thresholds for Experiment 1.

	Collinear 3λ	Collinear 4λ	Collinear 6λ	Collinear 8λ	Orthogonal 3λ	Orthogonal 4λ	Orthogonal 6λ	Orthogonal 8λ
AMD1	0.217	0.170	0.210	0.238	0.229	0.270	0.215	0.280
AMD2	0.486	0.316	0.193	0.230	0.242	0.257	0.211	0.282
AMD3	0.355	0.204	0.171	0.274	0.246	0.216	0.215	0.289
AMD4	0.122	0.177	0.164	0.110	0.071	0.121	0.221	0.143
AMD5	0.492	0.229	0.094	0.090	0.201	0.241	0.105	0.120
C1	0.201	0.078	0.048	0.056	0.065	0.056	0.071	0.101
C2	0.140	0.028	0.026	0.029	0.026	0.037	0.035	0.033
C3	0.371	0.413	0.141	0.104	0.208	0.171	0.209	0.129
C4	0.112	0.070	0.018	0.028	0.041	0.029	0.026	0.037
C5	0.180	0.093	0.056	0.060	0.067	0.095	0.134	0.124

Since the effect of the group was significant, we conducted separated analysis on MD participants and controls. In patients, a linear model ART with Orientation (Collinear *vs* Orthogonal) and Target-to-flankers distance (3λ, 4λ, 6λ, 8λ) as factors, showed a significant effect of Target-to-flankers distance (*F*_3,28_ = 3.11, *p* = 0.042). Post-hoc analysis showed a significant difference between 3λ and 6λ (p = 0.025).

In controls, a linear model ART with Orientation (Collinear *vs* Orthogonal) and Target-to-flankers distances (3λ, 4λ, 6λ, 8λ) as factors showed a significant main effect of Orientation (*F*_1,28_ = 6.06, *p* = 0.021) and Target-to-flankers distances (*F*_1,28_ = 14.642, *p* < 0.001).

Post-hoc analysis showed a significant difference between thresholds at 3λ and 6λ (*p* < 0.001) and between 3λ and 8λ (*p* < 0.001).

#### Threshold elevation

Following previous studies^17,21^, collinear inhibition and facilitation were computed as ratios of contrast thresholds (CT), using the following formula, separately for each target-to-flankers separation:

1 $$TE={\mathrm{log}}_{10}(\frac{CT\,collinear}{CT\,orthogonal})$$

We used the orthogonal condition rather than the Gabor alone as a baseline measure of facilitation to distinguish actual orientation-specific increase in contrast sensitivity from reduction of spatial uncertainty. This approach is standard for peripheral collinear facilitation measurements^19,21,35^.

For Experiment 1, we conducted a mixed model Aligned Rank Test on threshold elevations with between factor Group (MDs *vs* Controls) and within factor. Target-to-flankers distances (3λ, 4λ, 6λ, 8λ). The interaction was calculated using the r package npInt for Nonparametric Interaction Tests for Factorial Designs with Repeated Measures (Feys, 2015). Results showed a main effect of Target-to-flankers distance (*F*_3,24_ = 17.808, *p* < 0.001) and the interaction between factors (*F*_3,24_ = 5.39, *p* = 0.00554).

Post-hoc analysis (ART pairwise comparisons, holm adjusted) showed that overall threshold elevation at 3λ was significantly higher than all the other separations (*p* = 0.0391, *p* < 0.001 and *p* < 0.001 for 4λ, 6λ and 8λ, respectively). Additionally, we explored post-hoc tests for the interaction. Since tests of differences of combinations of levels between factors have issues in ART (Kay, 2014), it is recommendable to test for differences of differences; (i.e., for the interaction Group x Target-to-flankers distance, we might test whether the difference AMD - control is different for 3λ *vs* 4λ, Wobbrock *et al*., 2011). This analysis showed that the difference AMD-controls was significant between 3λ and 8λ (*p* = 0.014973) and 3λ and 6λ (*p* = 0.00193), suggesting that patients exhibited both less inhibition (0.213+/−0.156 *vs* 0.464+/−0.171) and less facilitation (−0.0836+/− 0.0391 *vs* −0.165+/−0.109) than controls at baseline.

#### MD patients

We estimated collinear facilitation by conducting a series of one sample t-tests (threshold elevation *vs* zero value) similarly to previous studies^20,21^. Results showed a significant threshold reduction for target-to-flankers separations of 6λ (*t*_4_ = 3.05, *p* = 0.038) and 8λ (*t*_4_ = 4.78, *p* = 0.009) and a significant threshold elevation for 3λ (*t*_4_ = 3.04, *p* = 0.039).

#### Controls

Independent one sample t-tests revealed a significant threshold reduction for target-to-flankers separations of 6λ (*t*_4_ = 4.78, *p* = 0.008) and 8λ (*t*_4_ = 3.38, *p* = 0.027) and a significant threshold elevation for 3λ (*t*_4_ = 6.04, *p* = 0.004).

Finally, comparing threshold elevation in the two groups, at 3λ MD patients showed less inhibition than controls (two-sample t-test, *t*_8_ = 0 2.42, *p* = 0.042), as well as lessr facilitation at 6λ (*t*_8_ = 1.17, *p* = 0.018).

### Training Results (Experiment 2)

#### Contrast thresholds

**Figure 6:**
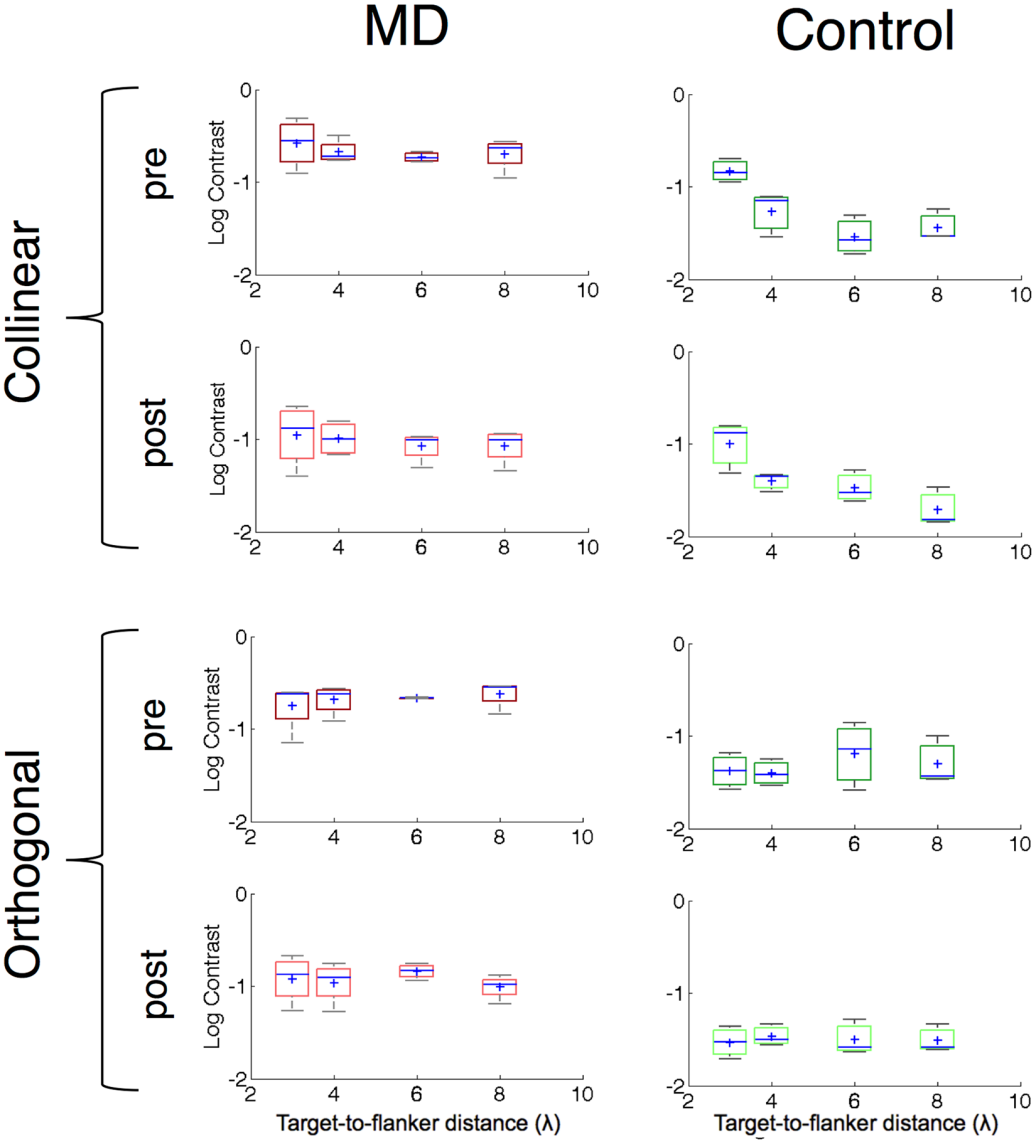
Box-and-whisker diagrams for collinear (above) and orthogonal (below) Michelson contrast thresholds for MD (red boxes) and Control participants (green boxes), before and after training as a function function of the target-to-flankers distances. See Fig. 5 for the details of the legend.

**Figure 7:**
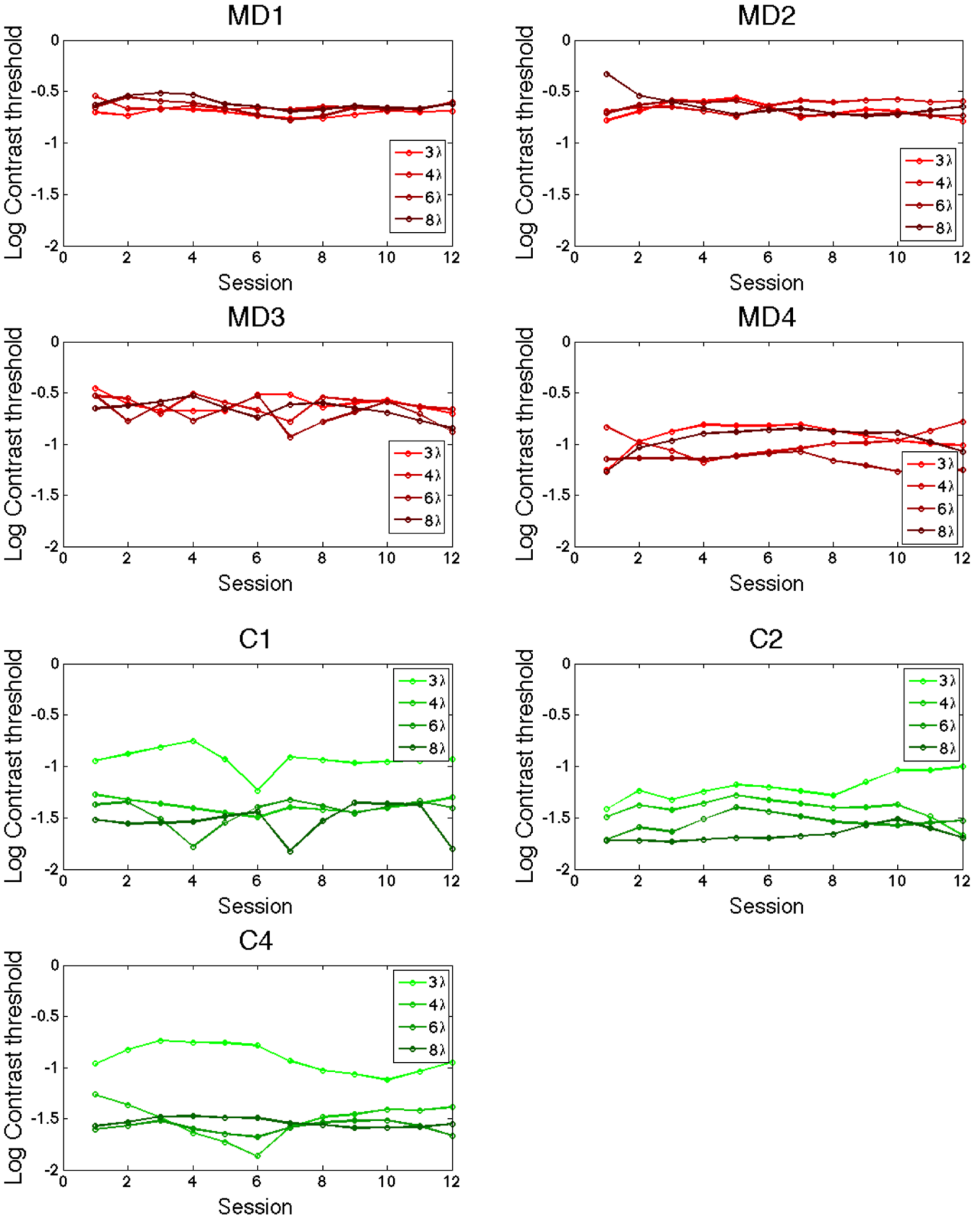
Single participant data for the training, divided by target-to-flankers distances (3 to 8 lambda, from lighter green to darker green for controls, from lighter red to darker red for MD patients).

For Experiment 2 (Fig. 6 and 7, Table 3), a Mixed Model ART with within factors Training (Pre *vs* Post), Orientation (Iso *vs* Ortho) and Target-to-flankers distances (3λ *vs* 4λ *vs* 6λ *vs* 8λ) and between factor group (MDs *vs* controls) showed a main effect of Group (*F*_1,5_ = 16.91, *p* = 0.009), Training (*F*_3,75_=121.44, *p* < 0.001) and Target-to-flankers distances (*F*_3,75_=5.57, *p* = 0.002): Again, it was not possible to run interaction tests. Post-hoc analysis showed that training significantly reduced contrast thresholds (*p* < 0.001). Since the between-subjects factor was significant, we conducted two separate ART for the two groups.

**Table 3:** Individual Michelson contrast thresholds difference after training (Experiment 2).

	Collinear 3λ	Collinear 4λ	Collinear 6λ	Collinear 8λ	Orthogonal 3λ	Orthogonal 4λ	Orthogonal 6λ	Orthogonal 8λ
AMD1	−0.122	−0.097	−0.107	−0.148	−0.115	−0.155	−0.101	−0.179
AMD2	−0.307	−0.181	−0.102	−0.121	−0.087	−0.081	−0.071	−0.176
AMD3	−0.130	−0.048	−0.065	−0.160	−0.034	−0.084	−0.057	−0.158
AMD4	−0.083	−0.108	−0.114	−0.064	−0.017	−0.068	−0.045	−0.078
C1	−0.092	0.017	−0.002	−0.014	−0.006	−0.006	−0.116	−0.007
C2	−0.070	−0.033	0.003	−0.022	−0.022	−0.009	−0.019	−0.054
C4	0.044	−0.040	0.011	−0.013	−0.012	−0.001	−0.001	−0.012

#### MD Patients

A Mixed Model ART on contrast thresholds with factors Training (Pre *vs* Post), Orientation (Iso *vs* Ortho) and Target-to-flankers distances (3λ *vs* 4λ *vs* 6λ *vs* 8λ) showed a main effect of Training (*F*_1,45_ = 88.69, *p* < 0.001).

#### Controls

A repeated on contrast thresholds with factors Training (pre *vs* post), Orientation (iso *vs* ortho) and Target-to-flankers distance (3λ *vs* 4λ *vs* 6λ *vs* 8λ) showed a main effect of Orientation (*F*_1,30_ = 9.07, *p* = 0.005) and Target-to-flankers distance (*F*_1,30_ = 9.43, *p* < 0.0001).

#### Threshold elevation

A mixed model ART with within factors Training (Pre *vs* Post) and target-to-flankers distances (3λ *vs* 4λ *vs* 6λ *vs* 8λ) and between factor Group (MD *vs* Control) showed a main effect of Target-to-flankers distances (*F*_3,35_ = 21.65, *p* < 0.001), a main effect of Group (*F*_1,5_ = 11.82, *p* = 0.018). The MD group showed lower threshold elevation (0.1+/−0.025 *vs* −0.041+/−0.021), indicating overall greater facilitation for all the separations tested. Moreover, paired t-tests comparing pre- and post- training performance separately for each target-to-flankers separation showed that controls worsened their performance at 6λ after training (*p* = 0.004), while pre- post- paired t-test in MD patients approached significance for 3λ (*p* = 0.062). However, control group showed low contrast thresholds for the large target-flanker distances, suggesting a small room for improvement during the training. This might have reduced the differences between collinear and orthogonal thresholds and could thereby be the cause of this apparent performance deterioration.

## Discussion

Cortical reorganization in patients with central vision loss, either spontaneous or training induced, is a topic of great interest in visual and clinical neuroscience. Recent results from psychophysics, neurophysiology and neuroimaging studies remain unclear. Indeed, previous studies using psychophysics approaches reported evidence for and against spontaneous cortical reorganization^4,6^, respectively. Similarly, some neuroimaging studies supported the hypothesis of a spontaneous neural reorganization in the lesion projection zone of MD patients^43^. This effect existed for stimuli presented outside the scotoma, around the PRL or not^44^. However, other fMRI studies did not find evidence for spontaneous cortical reorganization in MD patients^45^ or monkeys with retinal lesion^46^. In Experiment 1, we showed that in MD, both collinear inhibition and facilitation emerged at target-to-flankers separations that are consistent with those measured in previous studies in normal peripheral^19–21,35^ but not foveal^17^ vision. With respect to control participants, MD patients had overall higher contrast thresholds, and presented both reduced collinear inhibition at short separations (3λ) and reduced facilitation at larger separations (6λ and 8λ). On the one hand, reduced inhibition is consistent with the hypothesis of a spontaneous or use-dependent cortical reorganization. Previous training studies on collinear facilitation reported reduced inhibition after training^21,27^ suggesting neural plasticity in the early visual areas as possible neural substrate of this phenomenon^24–26^. On the other hand, the reduced facilitation and the emergence of the effect at target-to-flankers separations consistent with normal periphery rather than fovea might indicate that spontaneous cortical plasticity did not produce major reorganization in the PRL projecting zone, in agreement with recent psychophysical studies^5,6^. Overall, despite being tested at similar eccentricities, MD patients showed lower contrast sensitivity with respect to their age-matched controls. Previous studies on this population have shown that even in the absence of a clear lesion in the peripheral retina, a central scotoma might impair peripheral vision through a reduction of fixation stability and/or damages in the lateral connections of the retina^3^. Moreover, it has been shown that the PRL chosen by patients for fine vision and fixation might not always the most sensitive or adequate region of the spared retina^47,48^. Consistently, Van der Stigchel and colleagues^5^ showed that in a visual search task, AMD patients had inferior performances than controls tested with an artificial scotoma, suggesting that the switch from a fovea-based to a PRL-based reference frame might impair efficiency in a number of visual tasks.

### Spontaneous cortical reorganization and the size of the perceptual field in MD patients

As reported in the introduction, the border between collinear inhibition and facilitation can be used as an estimate of the PF size. Previous studies showed that the inhibitory region, and consequently the size of PF, increases with eccentricity^19,21^, suggesting a relationship with the cortical magnification factor. Measuring the size of PF might thus offer hints about the cortical reorganization processes in the MD brain, *i.e*., whether units formerly responding to foveal stimulation have been recruited to respond to stimuli around the PRL. Consequently, a reduction in size of the PF might be consistent with a large scale, spontaneous cortical reoganization. However, results from the present study does not seem to support this hypothesis, given that facilitation emerged at the same target-to-flankers distance for both patients and controls. Nonetheless, MD patients showed a significantly reduced inhibition at short separation, an effect associated with neural plasticity in peripheral collinear facilitation^21^. Future studies might train patients in their PRLs on shorter target-to-flankers separations with respect to those used here (<3λ), to test whether collinear inhibition (and consequently PF size) can be reduced to the spatial extent observed in the fovea. Succeeding in reducing facilitation at shorter separations than those used in the present study would actually reshape lateral interactions in the PRL in the same way as those of the healthy fovea.

### Training-induced cortical reorganization

Regarding training-induced cortical reorganization, the few existing studies report small or inconsistent improvements and limited transfer^8,10–12^. However, a recent training paradigm based on lateral interaction showed that patients improved in the trained task and learning transferred to visual acuity and, partially, to contrast sensitivity function (but not to crowding)^9^. In general, trainings based on lateral masking might be more effective because of the plasticity hypothesis and hierarchical mechanisms they rely upon^27^. In Experiment 2, we trained MD patients and controls for 12 sessions on the lateral interaction configuration. The rationale was that several rehabilitative approaches are based on similar training-induced modulation of collinear facilitation^14,16^. Results showed that MD patients (but not age matched controls) reduced their collinear inhibition at short flankers separations, to the point that on average, inhibition was no longer observed for all the separations tested. If the reduced inhibition observed in Experiment 1 is consistent with the hypothesis of spontaneous cortical reorganization, PL further amplified this reduction. This improvement is even larger than the one observed in younger, healthy participants^21^. Because the neural substrates of collinear facilitation lay in early visual cortex, a portion of which does not receive foveal input anymore, it is possible that a training specifically designed to stimulate these regions might further promote plasticity, e.g., by recruiting new neural units or by reshaping the PF size in a more fovea-like fashion. This result is particularly promising because the majority of rehabilitation approaches in MD patients currently focuses on improving reading speed and training-induced reduction of cortical inhibition is correlated with reduction of visual crowding^21^, one of factors contributing to slower letter recognition (and consequently reading speed) in peripheral vision^48^. However, one cannot rule out the possibility that low level neural plasticity is not the sole or main responsible of the observed performance changes. Other mechanisms, such as attentional modulation or read-out models, could also account for the training results^49,50^.

### Limitations of the study

Given the clinical population involved, the study presents a number of limitations. The main limitation of this study is the small number of patients. This small number is largely explained by the fact that PL trainings are often long. In our case, sessions extended over several weeks. It made it more difficult to recruit a large number of participants, and specifically of MD patients. However, several studies showed that reliable and robust results could still be obtained in this case by comparing perfomances before and after training^27,29–34^. In our study, patients consistently showed reduced inhibition with respect to controls at baseline as well as larger learning effect after training with respect to controls.

Additionally, we did not directly measure eye position (*e.g*., by imaging the retina or tracking the eye) during the training. Nonetheless, we used visual aids to facilitate fixation. This approach was shown to improve eye stability in previous studies on MD patients^38^. In our case, all the patients had a unique and well-defined PRL. Thus, they could not rely on a secondary PRL to perform the task. In addition, they all reported that both the flankers and the targets were visible througout the whole experiment. We are therefore confident that our results are very unlikely affected by fixation unstability. A direct measure of eye position would however have permitted to better characterize this aspect.

## Electronic supplementary material

Supplementary Figure
