# Supplementary material for: Spontaneous and training-induced cortical plasticity in MD patients: Hints from lateral masking
Source: Sci Rep. 2018 Jan 8;8:90. doi: 10.1038/s41598-017-18261-6 (PMC5758612; doi:10.1038/s41598-017-18261-6)
Supplement: Supplementary file 1 — Supplementary Figure [file 41598_2017_18261_MOESM1_ESM.pdf]

# Spontaneous and training-induced cortical plasticity in MD patients: Hints from lateral masking

Marcello Maniglia[1,2,31], Vincent Soler[4], Benoit Cottereau[2,32], Yves Trotter[2,32]

[1]UC Riverside, Riverside, California, USA

[2]Université de Toulouse-UPS, Centre de Recherche Cerveau et Cognition, Toulouse, France. [3]Centre National de la Recherche Scientifique, Toulouse Cedex, France.

[4]Unité de rétine, consultation d'ophtalmologie, hôpital Pierre-Paul-Riquet, CHU de Toulouse, place Baylac, 31059 Toulouse cedex, France

[1] Corresponding author: marcello.maniglia@ucr.edu
Department of Psychology, University of California Riverside, 900 University Ave., Riverside, CA 92521, USA
[2] Equal contribution last authors
**Keywords**: AMD, cortical plasticity, perceptual learning, peripheral vision

Supplementary Figures :

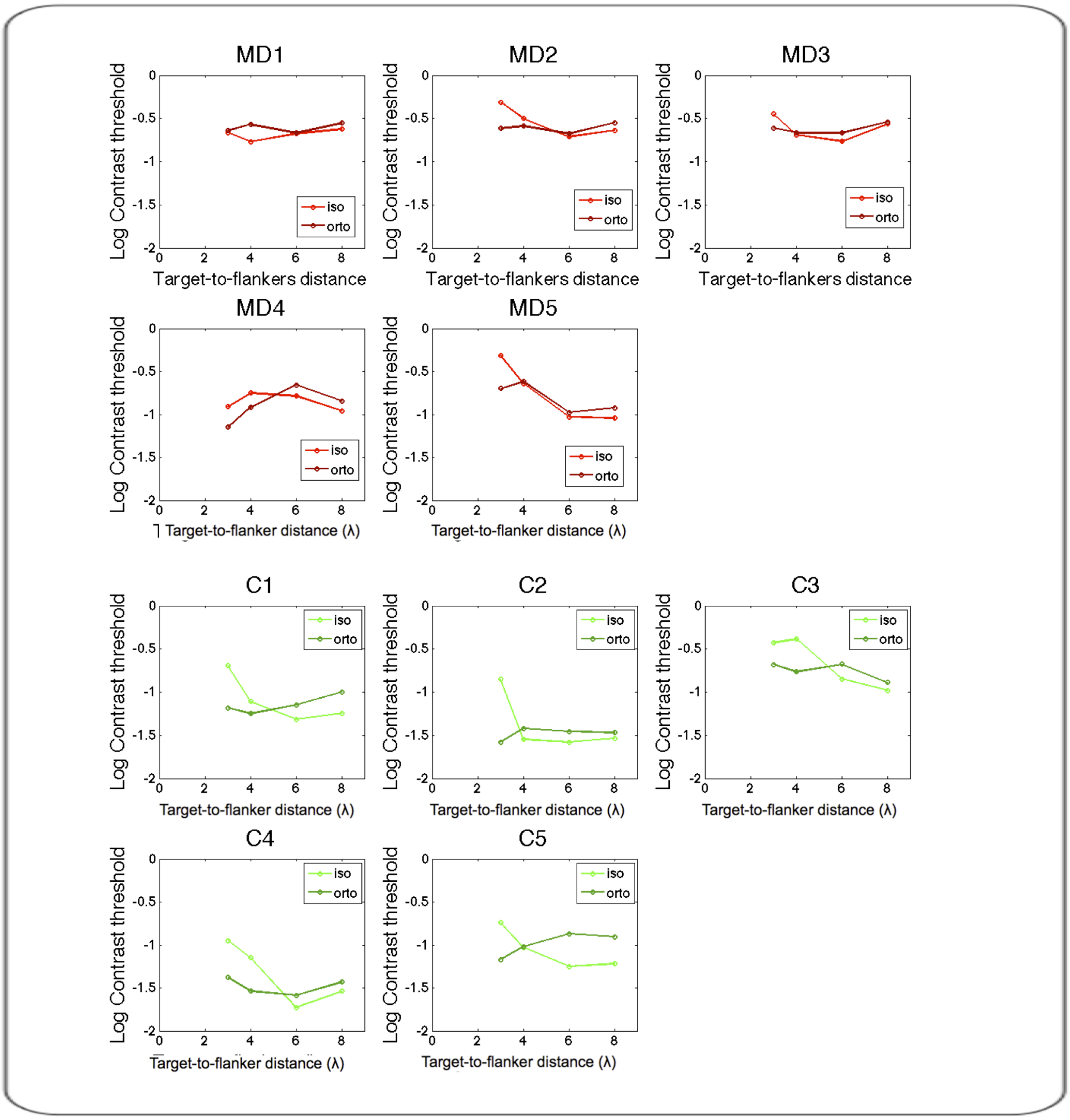


***Supp Figure 1** : Single participant data for Experiment 1, contrast thresholds (Michelson contrast) for the collinear (iso) and orthogonal (ortho) configuration for the 4 target-to-flankers distances tested.*
